# Long-Term Endovascular Management of a Large Superior Mesenteric Artery Aneurysm Secondary to Median Arcuate Ligament Syndrome: A Six-Year Follow-Up Study

**DOI:** 10.7759/cureus.88411

**Published:** 2025-07-21

**Authors:** Maciej Mach, Tomasz Ostrowski, Mikolaj M Rusin, Rafał Maciąg, Michał Sajdek, Zbigniew Gałązka

**Affiliations:** 1 Department of General, Vascular, Endocrine and Transplant Surgery, Medical University of Warsaw, Warsaw, POL; 2 Centre of Radiological Diagnostics, National Medical Institute of the Ministry of the Interior and Administration, Warsaw, POL; 3 2nd Department of Clinical Radiology, Medical University of Warsaw, Warsaw, POL

**Keywords:** dunbar syndrome, endovascular embolization treatment, median arcuate ligament syndrome, superior mesenteric artery aneurysm, visceral artery aneurysm

## Abstract

Median arcuate ligament syndrome (MALS), also known as Dunbar syndrome, is a rare vascular condition caused by compression of the celiac trunk by the median arcuate ligament, leading to altered visceral blood flow and promoting collateral circulation that can predispose patients to visceral artery aneurysms (VAAs), particularly within the pancreaticoduodenal arcade. These aneurysms carry a significant risk of rupture and require timely intervention. We present the case of a 75-year-old female patient with an incidentally discovered large superior mesenteric artery (SMA) aneurysm and complete celiac trunk occlusion due to MALS. The patient’s medical history was notable for multiple comorbidities and bilateral hip arthroplasties, which rendered magnetic resonance imaging (MRI) nonviable because of pronounced metal artifacts. Over a period of six years, she underwent several staged endovascular embolization procedures using detachable coils via various arterial access points. Despite initial technical success, imaging follow-up was compromised by artifacts from orthopedic implants and embolization materials, limiting precise assessment of the aneurysm. Residual perfusion and recanalization within the aneurysm sac necessitated repeated interventions. The patient declined surgical treatment, restricting therapeutic options and prompting continued conservative monitoring. This case illustrates the complexity of managing large visceral artery aneurysms in the context of MALS and altered vascular anatomy, particularly when imaging capabilities are limited. The inability to perform MRI significantly impacted optimal follow-up and required reliance on artifact-prone options such as Doppler ultrasound and computed tomography angiography (CTA). These limitations, coupled with the patient's refusal of surgery, highlight the importance of individualized treatment approaches, multidisciplinary collaboration, and transparent patient communication regarding therapeutic options, risks, and constraints. Endovascular management of complex SMA aneurysms associated with MALS is feasible but presents significant diagnostic and therapeutic challenges. Successful outcomes in such cases depend on careful procedural planning, selection of appropriate imaging techniques, and shared decision-making tailored to patient-specific anatomical and clinical factors.

## Introduction

Median arcuate ligament syndrome (MALS), also referred to as celiac trunk compression syndrome or Dunbar syndrome, is a rare vascular condition caused by the abnormal compression of the celiac trunk and surrounding neural structures by the median arcuate ligament, a connective tissue band of the diaphragm. This ligament typically forms an arch above the origin of the celiac trunk at the level of the T12 vertebra, but in some individuals, it lies lower than normal, resulting in compression of the celiac trunk [1]. The syndrome is uncommon, affecting approximately 2 out of every 100,000 people, and is seen more frequently in women between the ages of 20 and 50 [2]. Patients often present with nonspecific gastrointestinal symptoms such as postprandial epigastric pain, nausea, early satiety, vomiting, and unexplained weight loss [3]. These symptoms may result from impaired blood flow or irritation of the celiac plexus. Due to its nonspecific presentation, MALS is typically diagnosed accidentally. Surgical decompression of the celiac trunk, typically performed via open or laparoscopic release of the median arcuate ligament, remains the first-line treatment for MALS and should be considered whenever feasible [4]. Reduced blood flow through the celiac trunk in MALS cases can prompt the development of collateral circulation, particularly through the superior mesenteric artery [5]. The resulting increase in compensatory blood flow through collateral vessels can place abnormal hemodynamic stress on their walls, predisposing them to dilatation and the formation of true visceral artery aneurysms, especially within the pancreaticoduodenal arcade [6]. Though often asymptomatic and detected incidentally, these aneurysms can occasionally cause symptoms such as vague abdominal pain or, in more severe cases, gastrointestinal bleeding due to rupture [7]. The risk of rupture is significant even in small aneurysms, requiring early intervention. Management of MALS in the presence of visceral aneurysms poses a complex clinical challenge, with both endovascular and open surgical approaches available, yet no universally accepted treatment protocol exists.

We present the case of a 75-year-old female patient with a large superior mesenteric artery aneurysm secondary to MALS, managed over six years with multiple staged endovascular embolization procedures because of imaging limitations and the patient’s informed refusal of definitive surgical treatment, despite thorough counseling regarding an open therapeutic approach.

## Case presentation

A 75-year-old female patient was admitted to our department on February 20, 2019, for the management of an incidentally detected aneurysm of the superior mesenteric artery identified on ultrasound examination. At the time of admission, the patient was asymptomatic. She had a longstanding history of arterial hypertension, managed with nebivolol and amlodipine, and hypercholesterolemia, for which she was receiving rosuvastatin. Her past medical history included bilateral total hip arthroplasty and lumbar spine stabilization surgery. There was no history of COVID-19 infection; the patient had received three doses of the COVID-19 vaccine. Family history was notable for gastric cancer in the patient's father. The patient reported no history of hereditary or genetic disorders, diabetes mellitus, nicotine use, cerebrovascular events, or coronary artery disease. Physical examination revealed no abnormalities. Laboratory test results at admission are presented in Table 1.

**Table 1:** Laboratory test results on admission eGFR: estimated glomerular filtration rate, BUN: blood urea nitrogen.

Test	Result	Reference Range
Fibrinogen	337 mg/dL	200–400 mg/dL
Glucose	106 mg/dL	70–99 mg/dL
Creatinine	0.72 mg/dL	0.5–1.1 mg/dL
eGFR	82 mL/min	>60 mL/min
Urea (BUN)	27 mg/dL	15–48 mg/dL
Hemoglobin	14.3 g/dL	12–16 g/dL
Potassium	4.31 mmol/L	3.6–5.0 mmol/L
Sodium	142.7 mmol/L	137–145 mmol/L

**Figure 1:**
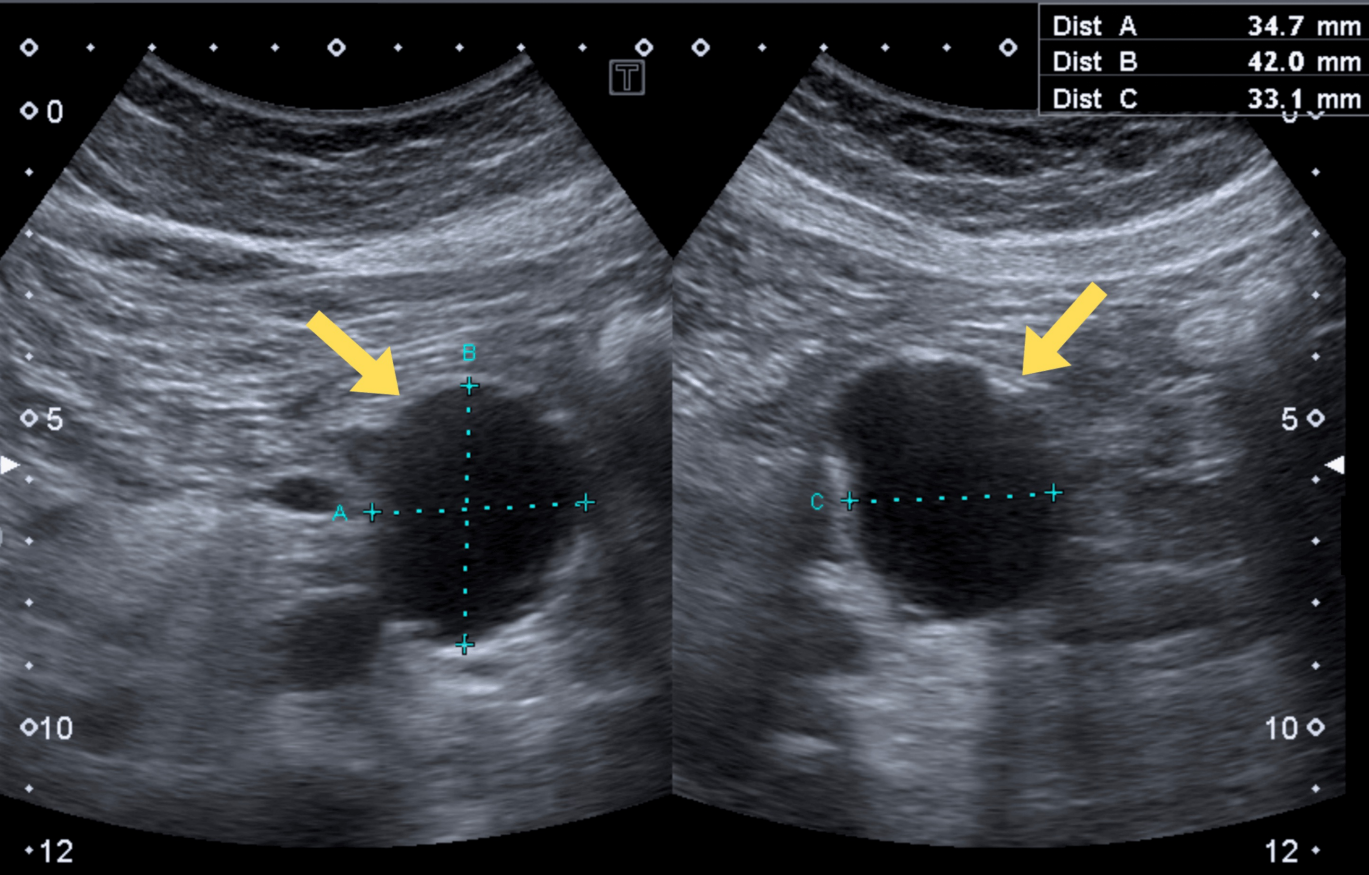
Ultrasound image showing an incidentally detected aneurysm. Yellow arrow indicates the superior mesenteric artery.

Initial abdominal and retroperitoneal ultrasound (USG) revealed an anechoic lesion measuring 35 × 42 × 33 mm, located below the body of the pancreas, with turbulent blood flow (Figure 1). The lesion was thought to most likely represent an aneurysm of the superior mesenteric artery and required further evaluation with computed tomography (CT) angiography.

**Figure 2:**
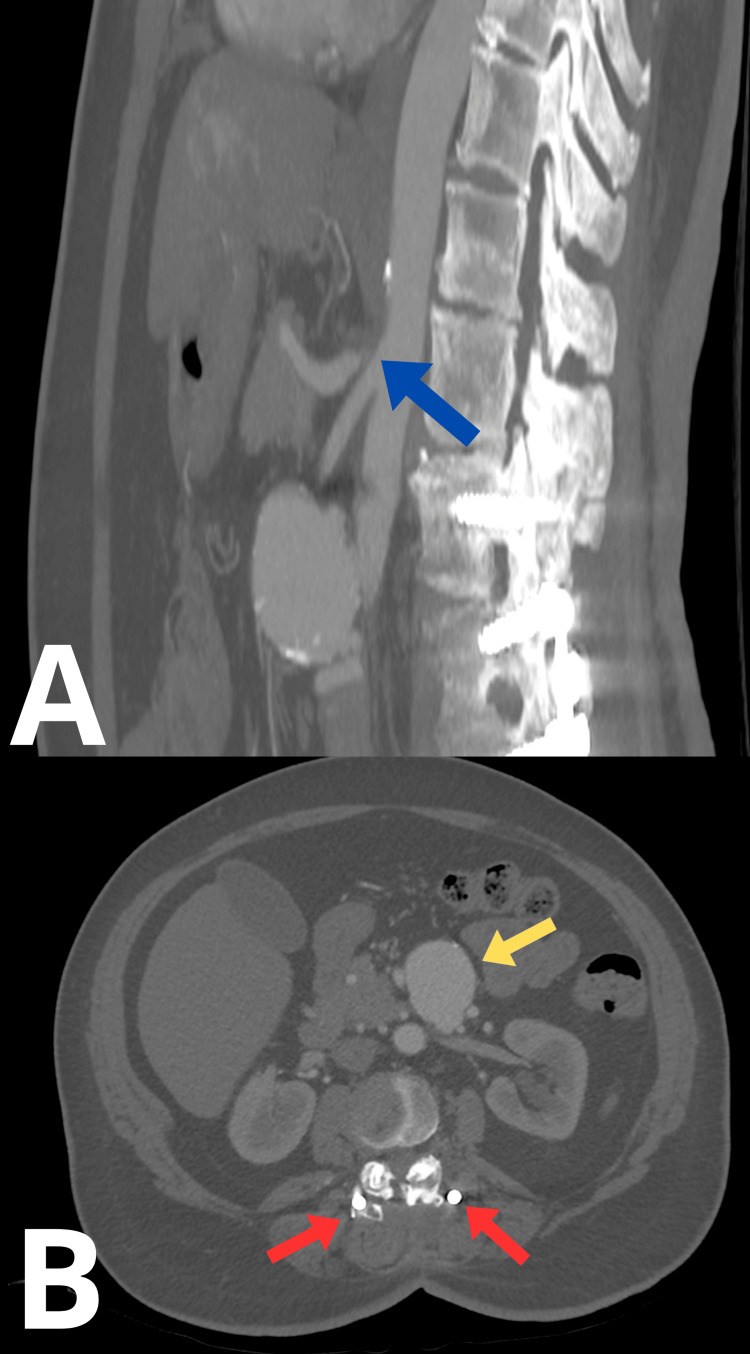
Initial CT scan. (A) Sagittal plane. (B) Transverse plane. The blue arrow indicates the compressed celiac trunk. The yellow arrow indicates the superior mesenteric artery aneurysm. The red arrow indicates spinal screws.

Contrast-enhanced abdominal CT revealed a complete occlusion of the celiac trunk (Figure 2A), as well as an aneurysmal sac of the superior mesenteric artery, located approximately 50 mm distal to the arterial ostium, measuring 47 × 36 mm in cross-section (Figure 2B). Image quality was partially limited due to significant artifacts from the bilateral hip prostheses. A decision was made to proceed with endovascular treatment as it allowed precise occlusion of pathological vessels with minimal surgical intervention, resulting in a lower risk of complications, shorter hospital stay, and faster return to daily activities, which was particularly important given the patient’s limitations and her refusal of surgical treatment. 

**Figure 3:**
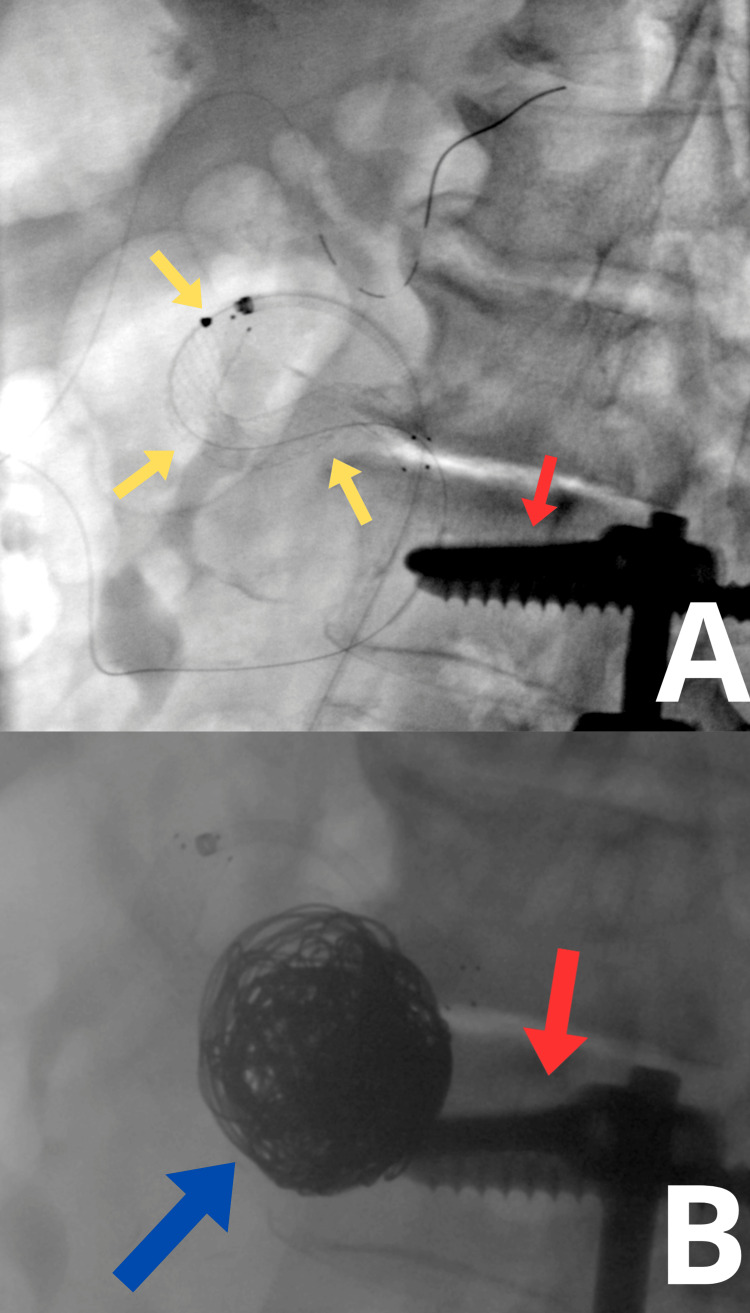
Intraoperative angio-CT. (A) Stent in the branch supplying blood to the liver. (B) Embolized aneurysm. The yellow arrow indicates the stent. The red arrow indicates spinal screws. The blue arrow indicates embolization coils.

Through an endovascular approach via the right axillary artery, a ZILVER 518 stent (8×60 mm) was implanted, securing the arterial outflow branch from the aneurysmal sac, which served as a major source of hepatic arterial blood supply (Figure 3A). Subsequently, embolization of the aneurysmal sac was performed using 17 detachable RUBY STANDARD coils (5 coils of 32 × 600 mm, 6 coils of 28 × 600 mm, and 6 coils of 24 × 570 mm), achieving satisfactory embolization (Figure 3B). During the procedure, 5,000 IU of intra-arterial heparin was administered. The endovascular access site was secured using an ANGIOSEAL 6F closure device. The postoperative course was uneventful, and the patient was discharged home on postoperative day 2.

**Figure 4:**
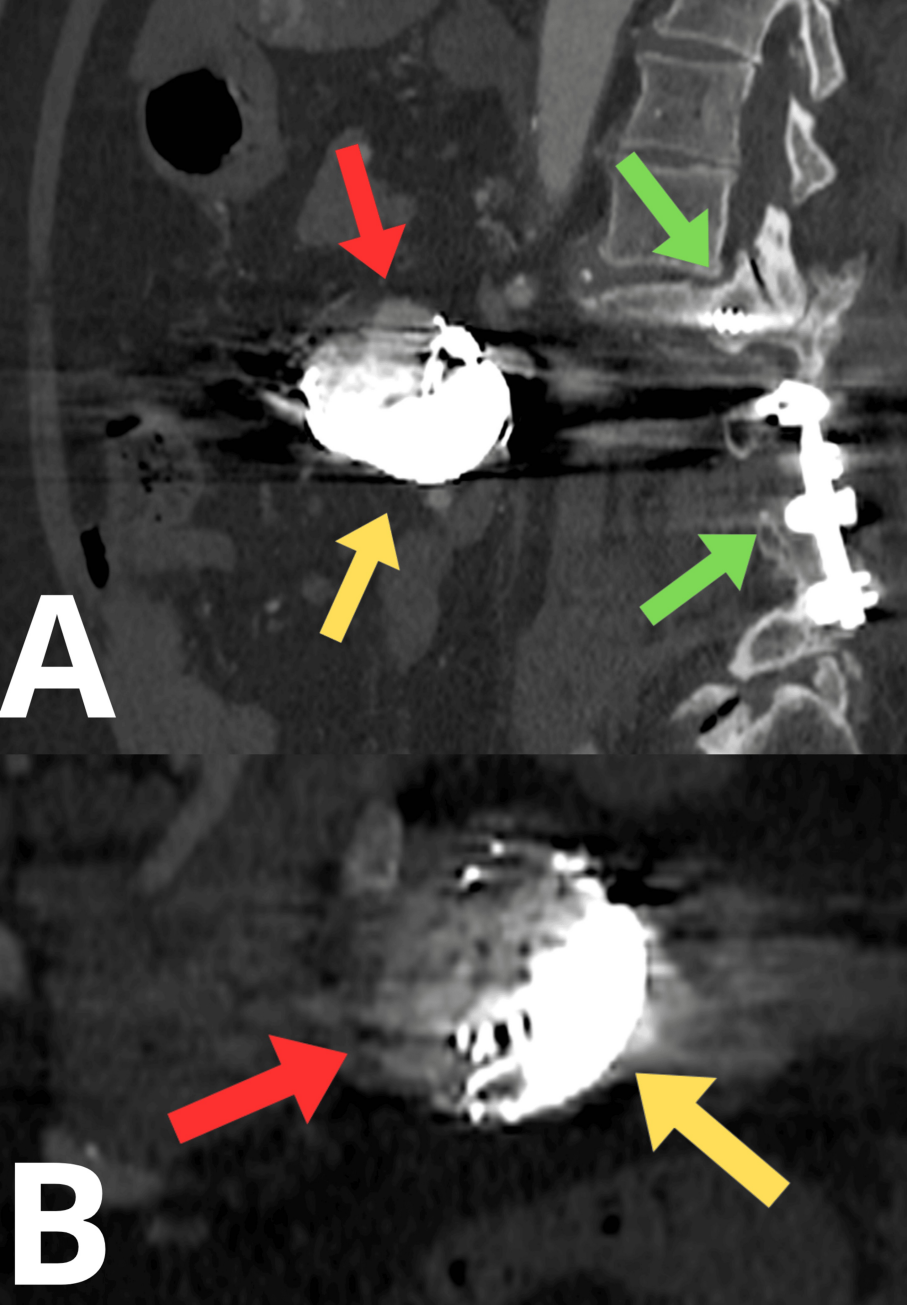
Angio-CT scan during follow-up. (A) Follow-up in April 2019. (B) Follow-up in February 2021. The yellow arrow indicates embolization coils. The red arrow indicates a filling aneurysmal sac. The green arrow indicates spinal screws and lumbar stabilization.

In April 2019, follow-up imaging was performed. CT angiography demonstrated an aorta without stenosis or aneurysmal changes, persistent occlusion of the celiac trunk, a patent superior mesenteric artery, and a satisfactory embolization effect within the first branch of the superior mesenteric artery (Figure 4A). The patient reported no symptoms. In follow-up CT angiography performed in February 2021, the embolized aneurysm measured approximately 50 × 38 × 39 mm, which was notably larger compared to the previous imaging (Figure 4B). Moreover, contrast enhancement within the aneurysmal sac was observed, indicating the presence of residual blood flow. No other abnormalities were detected.

**Figure 5:**
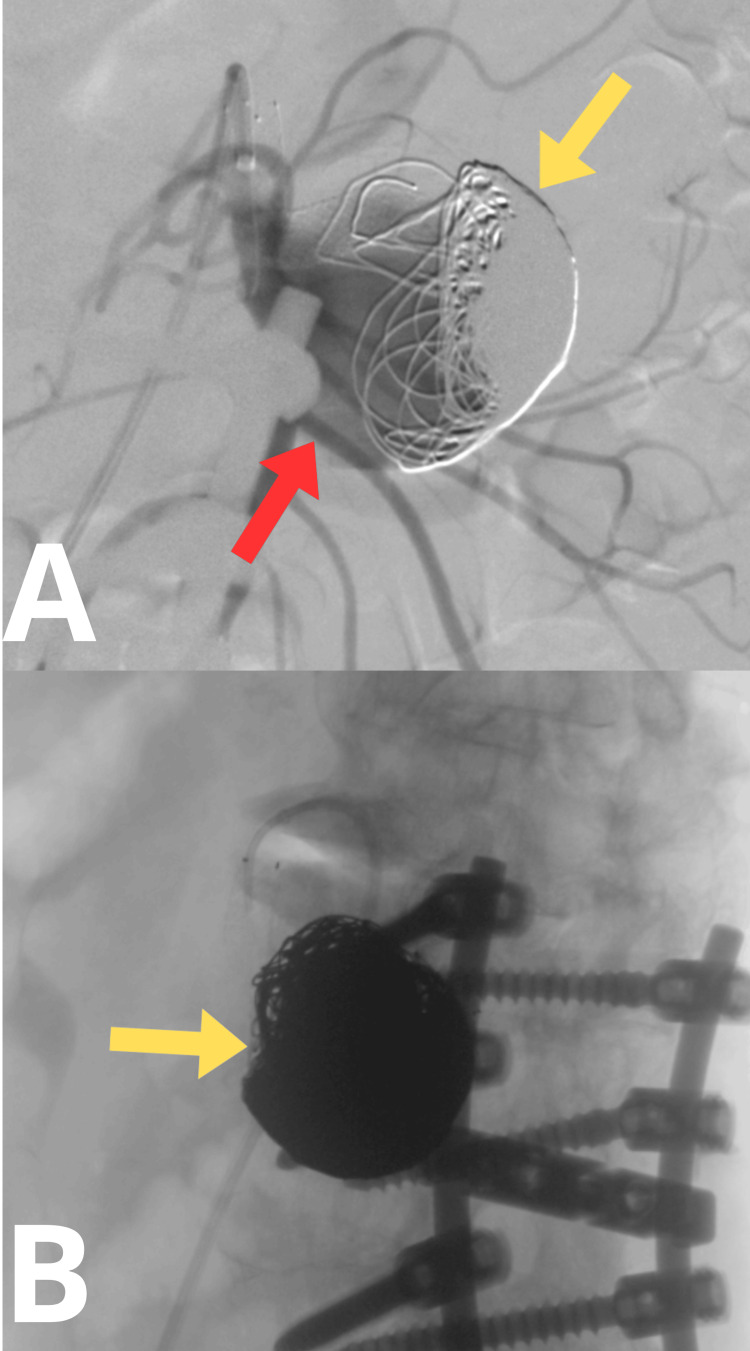
Intraoperative angio-CT. (A) Displaced embolization coils. (B) Complete aneurysm embolization. The yellow arrow indicates embolization coils. The red arrow indicates the filling portion of the aneurysmal sac.

In December 2021, a second stage of aneurysm embolization was scheduled. Selective arteriography of the superior mesenteric artery confirmed recanalization of the aneurysmal sac, with a characteristic water hammer effect: the sudden, pulsatile backward flow of blood caused by abrupt vessel occlusion or narrowing, creating a characteristic pressure wave visible during imaging, often indicating reflux or recanalization within an aneurysm or treated vessel (Figure 5A). During the same procedure, additional embolization of the aneurysmal sac was performed using 33 detachable embolization coils (RUBY STANDARD, RUBY SOFT, and PACKING COIL). Control arteriography confirmed the effectiveness of the procedure (Figure 5B). During the intervention, 10,000 IU of intra-arterial heparin was administered. The postoperative course was uneventful, and the patient was discharged home on postoperative day 2.

**Figure 6:**
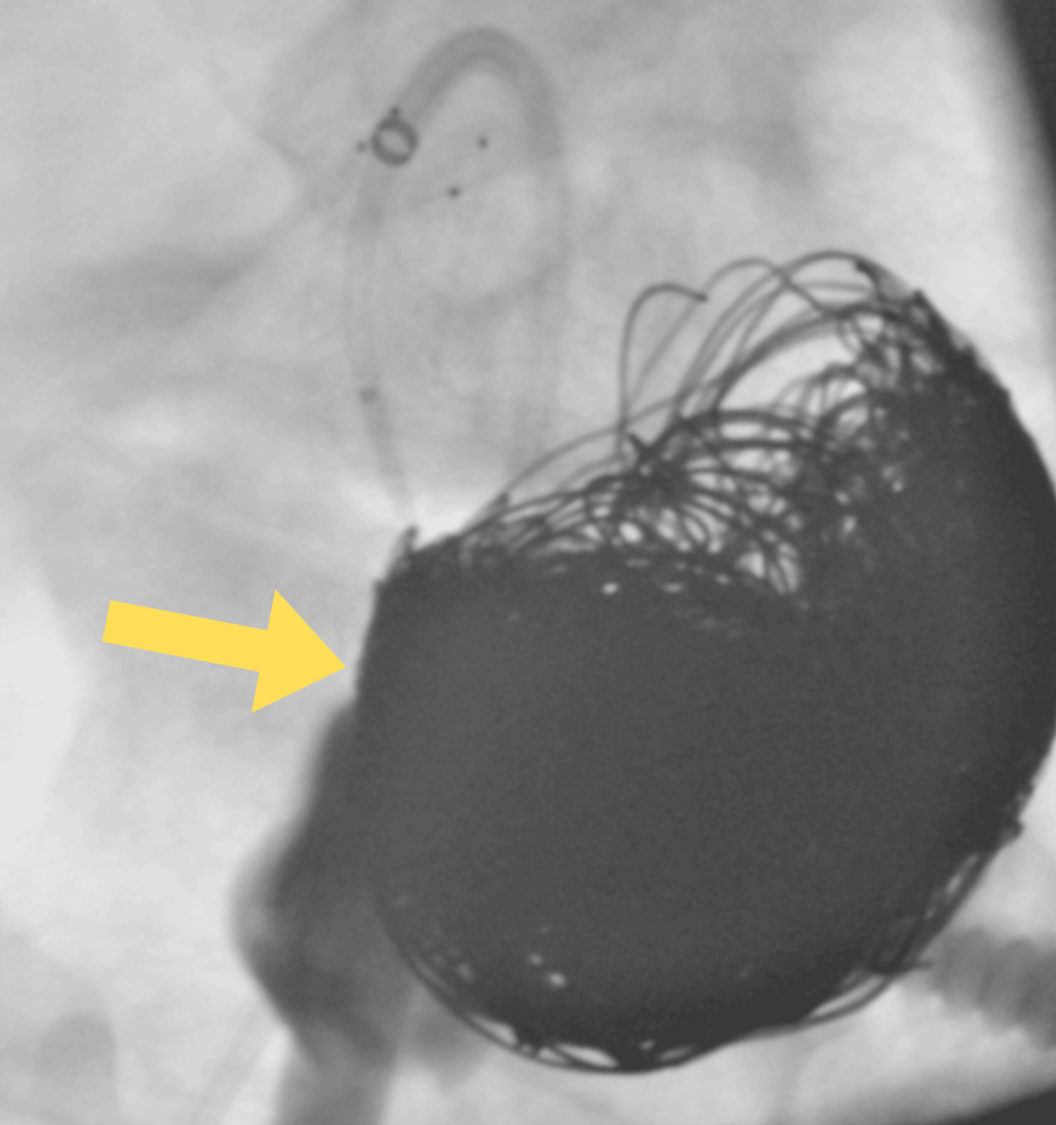
Intraoperative angio-CT, satisfactory embolization effect. The yellow arrow indicates embolization coils.

Follow-up CT angiography, performed in August 2022, demonstrated a persistent occlusion of the celiac trunk. The splenic artery and common hepatic artery remained patent and were supplied via collateral circulation. The embolized aneurysm measured approximately 55 × 44 mm in cross-section, with residual contrast filling observed in the upper portion of the aneurysm, measuring around 19 × 16 mm. The third stage of aneurysm embolization was scheduled for November 2022. Through a right femoral artery access, embolization of the potent part of the aneurysm was performed using 27 detachable embolization coils (POD and RUBY STANDARD), along with one free embolization coil (TORNADO 10/5 mm) (Figure 6). Control arteriography confirmed the effectiveness of the procedure. During the intervention, 5,000 IU of intra-arterial heparin was administered. The access site in the right groin was secured with an ANGIOSEAL 6F closure device. Control arteriography confirmed the effectiveness of the procedure. The postoperative course was uneventful, and the patient was discharged home on postoperative day 2.

**Figure 7:**
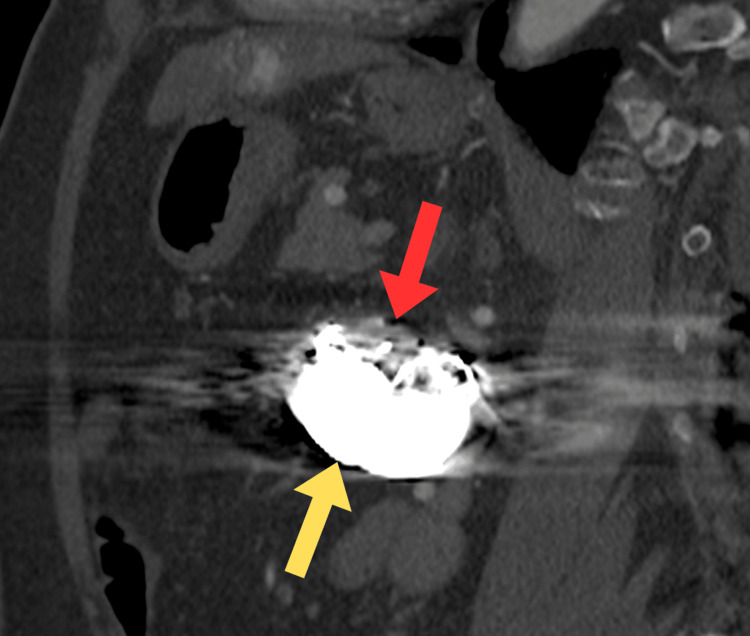
Angio-CT scan during follow-up. The yellow arrow indicates embolization coils. The red arrow indicates the trace filling portion of the aneurysmal sac.

Follow-up CT angiography performed in December 2023 demonstrated persistent occlusion of the celiac trunk, the splenic artery, and the common hepatic artery remained patent and were supplied via collateral circulation. Extensive artifacts from the embolization material significantly limited image assessment. The embolized aneurysm measured approximately 58 × 44 mm in cross-section, with residual contrast filling in the upper portion of the aneurysm, measuring approximately 20 × 15 mm, a reduction in size compared to the previous examination (Figure 7). Given the observed reduction in aneurysm size on this examination, a decision was made to abstain from further interventional treatment. 

**Figure 8:**
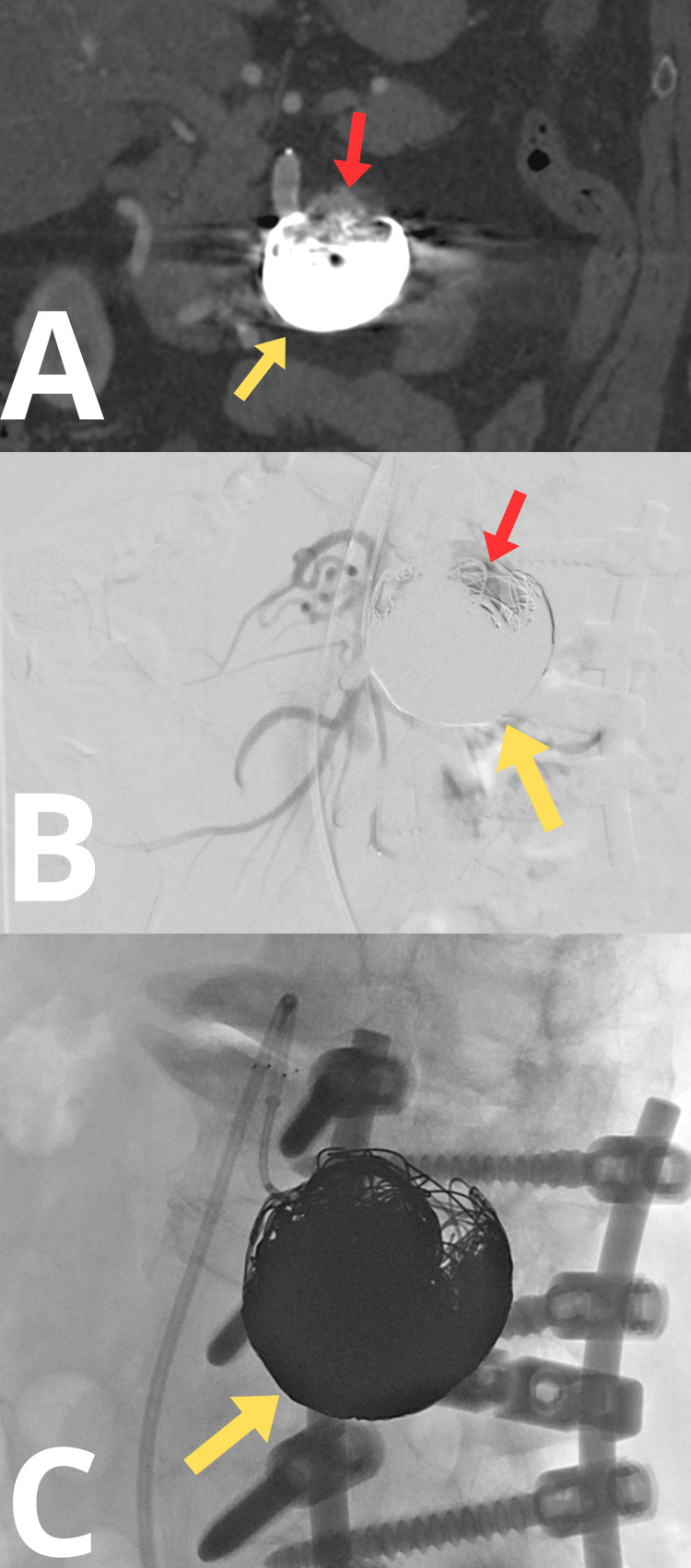
Angio-CT scans. (A) Visible contrast in the aneurysmal sac during the follow-up. (B) Intraoperative angio-CT. (C) Final embolization result. The yellow arrow indicates embolization coils. The red arrow indicates the trace filling portion of the aneurysmal sac.

In May 2025, CT angiography demonstrated an increase in aneurysm size, measuring 47 × 52 × 57 mm (Figure 8A). The 80-year-old patient declined surgical intervention, although she expressed concern about the risk of aneurysm rupture. In June 2025, the fourth stage of aneurysm embolization was performed. Through endovascular access in the left femoral artery, a complementary embolization of the aneurysm was carried out using 24 600-mm detachable embolization coils (RUBY STANDARD) (Figure 8B). Control arteriography confirmed the efficacy of the procedure (Figure 8C). During the intervention, 5,000 IU of heparin was administered intra-arterially. The access site in the left groin area was secured using a 6F ANGIOSEAL closure system. Postprocedural angiography demonstrated complete embolization of the aneurysmal sac, with no evidence of contrast opacification within the aneurysm, indicating successful exclusion from the arterial circulation. The superior mesenteric artery remained patent, and the overall result of the embolization was deemed fully satisfactory. The postoperative course was uneventful, and the patient was discharged home on postoperative day 2 in good clinical condition, free of symptoms, with plans for routine outpatient ultrasonographic monitoring.

## Discussion

MALS is a rare and frequently underdiagnosed condition characterized by chronic abdominal symptoms resulting from external compression of the celiac artery and adjacent neural structures by the median arcuate ligament [8, 9]. Although the exact etiology remains unclear, both vascular and neurogenic mechanisms are believed to contribute, namely ischemia due to reduced blood flow and irritation of the celiac plexus. The condition predominantly affects women between 30 and 50 years of age and is typically diagnosed by exclusion, as its clinical presentation often mimics other gastrointestinal or vascular disorders. Common symptoms include postprandial epigastric pain, nausea, vomiting, early satiety, diarrhea, and unintentional weight loss [8, 9]. Celiac artery stenosis caused by MALS has been strongly associated with the development of visceral artery aneurysms (VAAs), particularly in the pancreaticoduodenal region [10]. This relationship stems from the compensatory increase in blood flow through collateral vessels, such as the pancreaticoduodenal and gastroduodenal arteries, which leads to chronic hemodynamic stress, arterial wall weakening, and aneurysm formation [10]. Studies indicate that up to 24% of patients with MALS may develop splanchnic artery aneurysms, with pancreaticoduodenal aneurysms accounting for as many as 87.1% of cases [11]. These aneurysms pose a high risk of rupture regardless of size and are often diagnosed only after rupture has occurred, posing a serious mortality risk [12]. Prompt intervention is therefore essential, with endovascular embolization regarded as the treatment of choice due to its minimally invasive nature and lower complication rates compared to open surgery.

This case illustrates the considerable complexity of endovascular management in the context of MALS, particularly in a patient with a large SMA aneurysm and challenging vascular anatomy. Over a six-year period, the patient underwent multiple staged embolization procedures because of progressive aneurysm growth, recanalization, and persistent collateral circulation, maintaining perfusion of the aneurysmal sac. The therapeutic approach was further complicated by complete celiac trunk occlusion secondary to MALS, which significantly altered visceral arterial flow dynamics. Imaging follow-up was severely hindered by extensive artifacts from bilateral hip arthroplasties and previously placed embolization coils, preventing the use of MRI, the preferred modality for soft tissue evaluation, and necessitating reliance on artifact-prone techniques such as Doppler ultrasound and CT angiography. Although the embolization procedures were technically successful, residual perfusion and aneurysm enlargement persisted, highlighting the limitations of endovascular therapy in anatomically complex cases. Additionally, the patient’s refusal of surgical treatment further restricted management options, requiring conservative monitoring despite the ongoing risk of rupture.

## Conclusions

Endovascular interventions represent a valuable, minimally invasive option in the management of Dunbar syndrome with associated collateral vessel aneurysms, demonstrating favorable safety and efficacy profiles. However, this case illustrates the limitations of endovascular therapy in the setting of complex vascular anatomy, restricted imaging options because of orthopedic implants, and patient refusal of surgical intervention. The persistence of aneurysm growth despite multiple embolizations raises concerns regarding the long-term effectiveness of currently available embolic materials in such scenarios. These challenges emphasize the need for individualized treatment strategies, shared decision-making, and further research to clarify the thresholds at which surgical alternatives should be reconsidered.
